# Postoperative Opioid-Free Multistep Opiranserin Infusion Versus Fentanyl Patient-Controlled Analgesia After Breast or Minimally Invasive Gynecologic Surgery: A Retrospective Cohort Study

**DOI:** 10.3390/jcm15208062

**Published:** 2026-10-17

**Authors:** Yeojung Kim, Mia Park, Sungho Choi, Juyeon Kim, Jinwoo Kim, Yeonsu Kim, Seounghun Lee, Chan Noh, Jiho Park

**Affiliations:** 1 Department of Anesthesiology and Pain Medicine, Chungnam National University Sejong Hospital, Sejong 30099, Republic of Korea; 2 Department of Anesthesiology and Pain Medicine, Chungnam National University School of Medicine, Daejeon 35015, Republic of Korea; 3 Department of Obstetrics and Gynecology, Chungnam National University School of Medicine, Daejeon 35015, Republic of Korea; 4 Department of Obstetrics and Gynecology, Chungnam National University Hospital, 33 Munhwa-ro, Jung-gu, Daejeon 35015, Republic of Korea; 5 Department of Anesthesiology and Pain Medicine, Chungnam National University Hospital, Daejeon 35015, Republic of Korea; 6 Biomedical Research Institute, Chungnam National University Hospital, Daejeon 35015, Republic of Korea

**Keywords:** analgesia, patient-controlled, non-narcotic analgesics, opiranserin, postoperative pain, postoperative nausea and vomiting, retrospective studies

## Abstract

**Background/Objectives**: Women undergoing breast or gynecologic surgery face high postoperative nausea and vomiting (PONV) risk, which often prompts discontinuation of opioid patient-controlled analgesia (PCA). Since anticipated opioid use is an Apfel risk factor, we examined a postoperative opioid-free analgesic regimen using opiranserin, previously evaluated only as an adjunct. **Methods**: In this single-center retrospective cohort study, 150 women undergoing breast or minimally invasive gynecologic surgery received a multistep opiranserin infusion via a PCA pump without demand boluses (*n* = 77) or fentanyl demand bolus PCA (*n* = 73). The opiranserin infusion ended at 10 h; fentanyl PCA remained connected throughout. The primary outcome was resting numeric rating scale (NRS, 0–10) at 24 h, by which time the infusion had been complete for 14–15 h; secondary outcomes were post-anesthesia care unit (PACU) and 6 h NRS, PONV, and early discontinuation. **Results**: Resting NRS did not differ significantly during the infusion (PACU *p* = 0.965; 6 h *p* = 0.675), with a larger difference at 24 h. The adjusted 24 h difference was 0.58 points higher with opiranserin (95% CI, 0.04 to 1.12; *p* = 0.036) by covariance analysis on pre-treatment covariates and 0.55 (0.02 to 1.09) by inverse-probability-of-treatment weighting; the point estimates were below the minimal clinically important difference of 1 point, but the confidence intervals do not exclude a clinically relevant increase in pain, and the estimate was nonsignificant after adjusting for non-opioid adjuncts (*p* = 0.177). Moderate or severe pain at 24 h was more frequent with opiranserin (19.5% vs. 8.2%; adjusted odds ratio, 3.76; 95% CI, 1.34 to 11.82). PONV (9.1% vs. 11.0%), rescue analgesia (26.0% vs. 17.8%), and early discontinuation (10.4% vs. 9.6%) were similar. Rescue opioids were available to both groups but used by one fentanyl-group patient and by none receiving opiranserin. **Conclusions**: The regimen eliminated postoperative opioid exposure, with resting pain scores that differed by less than 1 NRS point on average, although a clinically relevant increase cannot be excluded. With coverage fixed at 10 h, the 24 h difference cannot be separated into a shortfall of the agent and a shortfall of coverage. The anticipated PONV reduction was not observed, possibly in the context of low event rates and routine antiemetic prophylaxis.

## 1. Introduction

Women undergoing breast or gynecologic surgery present a distinct challenge in postoperative pain management. They are among the patients at highest risk of postoperative nausea and vomiting (PONV), since female sex and non-smoking status alone confer two of the four Apfel risk factors [1], yet they are also the patients to whom opioid-based intravenous patient-controlled analgesia (PCA) is routinely offered. When nausea develops, the analgesic device itself is often the first thing to be stopped, so that the treatment intended to control pain is abandoned because of its own adverse effect.

The fourth Apfel component is anticipated postoperative opioid use [1]. Removing the opioid from the PCA reservoir therefore removes one of the four risk factors outright, and this rationale has driven interest in opioid-free strategies in precisely this population [2,3,4]. Multimodal analgesia is already recommended to limit opioid exposure [5,6], but in practice the opioid component has proved difficult to eliminate entirely because the available non-opioid agents act through a narrow range of mechanisms and are often insufficient on their own [7]. We previously found that nefopam alone within a PCA did not achieve non-inferiority to fentanyl in this population and concluded that a multimodal rather than single-agent approach would be required [8].

Opiranserin (VVZ-149) is a first-in-class analgesic that simultaneously inhibits glycine transporter type 2 and the serotonin 5-HT2A receptor, modulating nociceptive transmission at the spinal dorsal horn through a mechanism distinct from that of opioids and non-steroidal anti-inflammatory drugs [9]. In phase 2 and phase 3 trials, it reduced pain intensity and lowered opioid consumption by approximately 30% after laparoscopic colectomy [10], laparoscopic colorectal resection [11], and laparoscopic or robot-assisted gastrectomy [12], and it was approved in Korea in December 2024 for moderate-to-severe acute postoperative pain [9]. In every one of those trials, however, the drug was given as an addition to opioid PCA: patients in both arms retained access to fentanyl PCA and to rescue opioids, and the question was how much opioid the drug could spare [10,11,12]. Whether opiranserin can serve as the analgesic base of a regimen containing no opioid at all has not been examined. The trials published to date have been confined to abdominal visceral surgery.

Since November 2025, our institution has offered opiranserin as a programmed multistep infusion (a 30 min loading dose followed by 9.5 h of maintenance) delivered through a PCA pump, with the demand bolus function disabled and no opioid in the reservoir, as an alternative to conventional fentanyl PCA in women undergoing breast-conserving or minimally invasive gynecologic surgery. We expected that a postoperative opioid-free approach would reduce PONV and thereby allow the analgesic device to be continued.

We therefore reviewed the first six months of this protocol to determine whether a postoperative opioid-free regimen based on opiranserin could be implemented without a substantial increase in postoperative pain compared with fentanyl PCA and whether it would reduce PONV in this population.

## 2. Materials and Methods

### 2.1. Study Design and Ethics

This single-center retrospective cohort study was approved by the Institutional Review Board of Sejong Chungnam National University Hospital (CNUSH 2026-05-001; approved 10 May 2026), which waived the requirement for written informed consent because only de-identified records of care that had already been completed were analyzed. The protocol, including the primary endpoint and a target sample size of 150 patients, was finalized on 30 April 2026, and no data were extracted from the electronic medical record before Institutional Review Board approval. The study was conducted in accordance with the Declaration of Helsinki and is reported according to the STROBE statement [13].

### 2.2. Patients

We screened women aged 19 years or older who underwent general anesthesia at our institution between 15 November 2025 and 30 April 2026 for breast surgery or minimally invasive gynecologic surgery (laparoscopic or robot-assisted) for benign gynecologic disease, and who received intravenous PCA postoperatively. All eligible patients treated during the study interval were screened consecutively, without interruption or selection. Because definitive pathology was in many cases established intraoperatively by frozen section, the extent of breast resection was not fixed before surgery; the cohort is therefore defined by the procedure performed rather than by preoperative indication.

Patients were excluded for American Society of Anesthesiologists (ASA) physical status IV or higher, chronic preoperative opioid use, ongoing treatment for chronic pain, missing or unevaluable records within 24 h after initiation of analgesia, or more than one concurrent PCA prescription.

Records meeting these criteria were further screened for factors that would preclude a valid comparison. We excluded records in which no PCA main agent was used or documented, solutions in which nefopam had been admixed, and cases in which a non-standard intraoperative opioid regimen was used, namely sufentanil instead of remifentanil or omission of remifentanil with administration of intraoperative fentanyl. When a patient underwent repeat surgery during the study period, only the first operation was retained to avoid carry-over of pain sensitization and prior opioid exposure. On review of the operative records, we additionally excluded patients who had undergone mastectomy with immediate breast reconstruction, a substantially more extensive procedure than the breast operations the approved study protocol was designed to capture.

Because opiranserin was administered under its approved labeling, patients scheduled for the opiranserin regimen were separately screened for label-specified contraindications. A 12-lead electrocardiogram is obtained routinely before surgery at our institution; patients with a QRS interval > 200 ms or QTcF > 470 ms, unstable angina, or congestive heart failure were not eligible, as were those with moderate-to-severe hepatic or renal impairment, pregnancy or lactation, or concurrent use of strong CYP3A4 inhibitors or drugs prolonging the PR or QRS interval.

### 2.3. Treatment Allocation

The postoperative analgesic regimen was determined at the preanesthetic assessment. An anesthesiology resident explained the opiranserin-based regimen to eligible patients using a standardized information leaflet, noting that neither regimen is reimbursed by the Korean National Health Insurance, that the opiranserin regimen is the more expensive of the two, and that conventional opioid PCA provides adequate analgesia so that declining opiranserin would not compromise postoperative pain control. Written consent for out-of-pocket payment was obtained from patients who agreed to receive it, and those patients were scheduled for the opiranserin regimen.

Because opiranserin had been approved only recently and no prior evidence was available on its use as a replacement for opioid PCA, the anesthesiology and surgical teams jointly reviewed each case, and conventional fentanyl PCA was selected when a larger or more painful procedure was anticipated. Allocation therefore reflected the patient’s consent together with a multidisciplinary judgement of expected pain severity.

### 2.4. Anesthesia

On arrival in the operating room, standard monitoring was applied, including noninvasive blood pressure measurement, pulse oximetry, electrocardiography, and processed electroencephalography with either the Bispectral Index (Medtronic, Minneapolis, MN, USA) or the SedLine sensor (Masimo, Irvine, CA, USA). Anesthesia was induced with propofol (1–2 mg/kg) and rocuronium (0.6 mg/kg), and tracheal intubation was performed 90 s later. Anesthesia was maintained with sevoflurane or desflurane at the attending anesthesiologist’s discretion under processed electroencephalographic guidance. Intraoperative opioid was administered as remifentanil by target-controlled infusion at an effect-site concentration of 2–3 ng/mL in both groups; the total dose was converted to µg using the institutional dilution of 50 µg/mL; the term opioid-free therefore refers to the postoperative period only. Core temperature was monitored with a zero-heat-flux cutaneous sensor (3M Bair Hugger Temperature Monitoring System; Solventum, Maplewood, MN, USA). At the end of surgery, all anesthetic agents were discontinued and neuromuscular blockade was reversed with sugammadex (200 mg intravenously). No patient received a regional or neuraxial block. Prophylactic antiemetics were administered at the anesthesiologist’s discretion and were not standardized between groups; palonosetron, ramosetron, and dexamethasone were used in one or more agents per patient [14].

### 2.5. Analgesic Regimens

Both regimens were delivered by the same electronic infusion pump (Accumate 1200; Woo Young Medical, Jincheon, Republic of Korea), which provides several infusion modes with a stated flow-rate accuracy of ±5% and stores up to 2000 device events that can be downloaded to a personal computer. The two groups therefore differed not in the device but in the infusion mode, the drug, and the timing of initiation.

The opiranserin group received a multistep infusion prepared according to the approved labeling: opiranserin 1000 mg (100 mL) diluted in 400 mL of normal saline to a total volume of 500 mL. Using the multistep mode, the pump delivered 160 mL/h for 30 min followed by 44.2 mL/h for 9.5 h, giving a fixed total duration of 10 h. The patient-demand bolus function was disabled, and the solution contained no opioid. Ramosetron (2 ampoules) were admixed in the infusate in most patients, a practice adopted in January 2026. Because its duration was fixed at 10 h, analgesic coverage ended approximately 9 h after the end of surgery in a typical case, given a median operation time of 55 min.

The fentanyl group received conventional demand bolus PCA containing fentanyl (1500 µg) with ramosetron (2 ampoules) in a total volume of 100 mL, delivered in the PCA mode of the same pump. The device provided a demand bolus of a median of 15 µg (range, 10–20) with a median lockout interval of 10 min (range, 10–20).

In the opiranserin group, the infusion was started after anesthetic induction and before skin incision, and the concomitant non-opioid analgesic was given as wound closure began. In the fentanyl group, non-opioid analgesics were administered during surgery, and the PCA device was connected at the start of wound closure, when a loading dose of fentanyl (0.5 µg/kg) was given. The two regimens therefore began at different points: opiranserin before incision and fentanyl PCA towards the end of surgery.

Concomitant systemic non-opioid analgesics, principally nefopam, acetaminophen, and a fixed-dose acetaminophen–ibuprofen combination, were not standardized between groups. In the opiranserin group, nefopam was routinely paired with the infusion, having been selected because, unlike non-steroidal anti-inflammatory drugs, it does not impair platelet function or renal prostaglandin synthesis, and unlike opioids, it does not depress ventilation [15,16]; in the fentanyl group, the choice of adjunct followed the preference of the attending anesthesiologist assigned to that operating room. Rescue analgesics and rescue antiemetics were administered on the ward as needed according to local protocol, and first-line agents differed by surgical department. Ward prescribing was not restricted by the study protocol, and rescue opioids remained available to patients in either group who had breakthrough pain that required them.

### 2.6. Outcome Measures

The primary outcome was resting pain intensity on an 11-point numeric rating scale (NRS, 0–10) at 24 h after surgery, taken as the nursing-recorded resting score closest to 24 h within a 3 h window. This time point was prespecified in the study protocol for three reasons: it corresponds to the routine 24 h nursing pain assessment at our institution and was therefore the measurement most consistently documented and retrievable for retrospective review; pain intensity at rest is a core outcome domain for perioperative pain management [17] and is commonly assessed on the first postoperative day across surgical procedures [18] so that a 24 h assessment permits comparison with previous work [10,19]; and it reflects the period most relevant to early recovery, ambulation, and sleep. The fixed 10 h opiranserin infusion had, however, ended well before this assessment so that the primary comparison necessarily falls outside the pharmacological coverage of that regimen.

Secondary outcomes were resting NRS on arrival in the post-anesthesia care unit (PACU) and at 6 h, both of which fall within the period of opiranserin coverage; PONV within 24 h, defined as at least one nursing record of nausea or vomiting or at least one administration of a rescue antiemetic; the numbers of rescue analgesic and rescue antiemetic doses within 24 h; rescue analgesic use classified by drug class; and early discontinuation of the analgesic device together with the recorded reason. Pump event logs were transferred automatically to the electronic medical record until this interface failed in mid-December 2025; the failure was identified only during data cleaning for this study, and logs generated after that date were not retained and could not be recovered. In the subset of fentanyl-group patients whose pump event logs could be retrieved, cumulative fentanyl delivered and the ratio of delivered to attempted boluses were described. Cumulative dose was read from the cumulative-amount field of the pump log and interpolated at 12 and 24 h from the start of pump operation, inclusive of the loading dose; where a power cycle reset the device counter, the record was truncated at that point; and where the pump was stopped before 24 h, the value at discontinuation was carried forward. Exactly duplicated event rows, which were present in five of the exported files, were removed before counting. Bolus attempts and deliveries were counted from individual device events within 24 h. Every log file was verified against the case report form by matching the patient identifier stored in the device record. Secondary analyses were exploratory and were not adjusted for multiplicity.

### 2.7. Statistical Analysis

Assuming a between-group difference of 1 NRS point, the reported minimal clinically important difference (MCID) for acute postoperative pain [20], with a standard deviation of 2 points, a two-sided alpha of 0.05, 80% power, and 1:1 allocation, 64 patients per group were required. Allowing for approximately 15% data loss inherent to retrospective record review, a target of 75 patients per group was set.

All statistical analyses were performed using Python version 3.12.3 (Python Software Foundation, Wilmington, DE, USA) with the pandas (version 3.0.2), NumPy (version 2.4.4), SciPy (version 1.17.1), and statsmodels (version 0.14.6) packages. All tests were two-sided, and *p* < 0.05 was considered significant. The distribution of each continuous variable was examined within each group using the Shapiro–Wilk test; every variable departed from normality in at least one group, so continuous variables are presented as median (interquartile range) and were compared using the Mann–Whitney U test. Categorical variables are presented as number (percentage) and were compared using the Fisher exact test. For the principal binary outcomes, risk differences are reported with Newcombe hybrid score confidence intervals, and adjusted odds ratios were estimated by Firth penalized logistic regression with profile penalized-likelihood confidence intervals, with penalization being used because of the small number of events. Baseline imbalance was quantified using the standardized mean difference (SMD), with an absolute SMD < 0.1 regarded as negligible. The analysis code was written with the assistance of a generative AI tool (Claude, Anthropic PBC, San Francisco, CA, USA; https://claude.ai, accessed 30 September 2026, see Acknowledgments). The choice of models, covariates and sensitivity analyses, and the interpretation of all results, were made by the authors, and the statistical analysis was independently verified by a co-author (Y.S.K.).

The causal contrast of interest is between an opiranserin-based postoperative multimodal regimen and a fentanyl-based postoperative multimodal regimen, not between opiranserin and fentanyl as single agents. The between-group difference in 24 h NRS was estimated by analysis of covariance (ANCOVA), adjusting for pre-treatment covariates only: age, body mass index, ASA physical status, hypertension, diabetes mellitus, thyroid disease, and planned procedure type. Operation time and intraoperative remifentanil dose are determined after treatment allocation, since opiranserin was started before skin incision, and were therefore treated as potential mediators rather than as baseline confounders. Four sensitivity models were fitted: one additionally including operation time and intraoperative remifentanil dose, one additionally including concomitant non-opioid analgesics, and two that varied the cohort definition. Inverse-probability-of-treatment weighting (IPTW) used a propensity score for receiving opiranserin estimated by logistic regression on the same pre-treatment covariates [21], with stabilized weights truncated at the 99th percentile; covariate balance before and after weighting was assessed by SMD; and weight distribution, propensity-score overlap, and the effective sample size after weighting are reported. Because pain was measured repeatedly in the same patients, a linear mixed-effects model with a random patient intercept and a generalized estimating equation with an exchangeable working correlation were also fitted, each including group, time, a group-by-time interaction, and the same pre-treatment covariates. Heteroscedasticity-consistent (HC3) robust standard errors were applied to the covariance-adjusted and weighted linear models, and IPTW estimates were verified by bootstrap resampling with 2000 replicates, including re-estimation of the propensity score.

Because the NRS distribution showed a pronounced floor effect, a proportional odds ordinal logistic model was fitted as a distributional check. The Apfel simplified risk score was not used in its recorded form, since one of its four components is determined by group assignment rather than by a pre-treatment characteristic [1]. The adjusted difference in 24 h NRS and its confidence interval were referenced against an MCID of 1 point [20]; because no equivalence or non-inferiority margin was specified in advance, and because the sample size was calculated within a conventional superiority framework, no claim of non-inferiority or equivalence can be made and this comparison is descriptive and post hoc. Distributional checks were performed with the assistance of a generative AI tool (Claude, Anthropic); all results were verified by the authors against the primary dataset.

## 3. Results

### 3.1. Study Population and Baseline Characteristics

**Figure 1:**
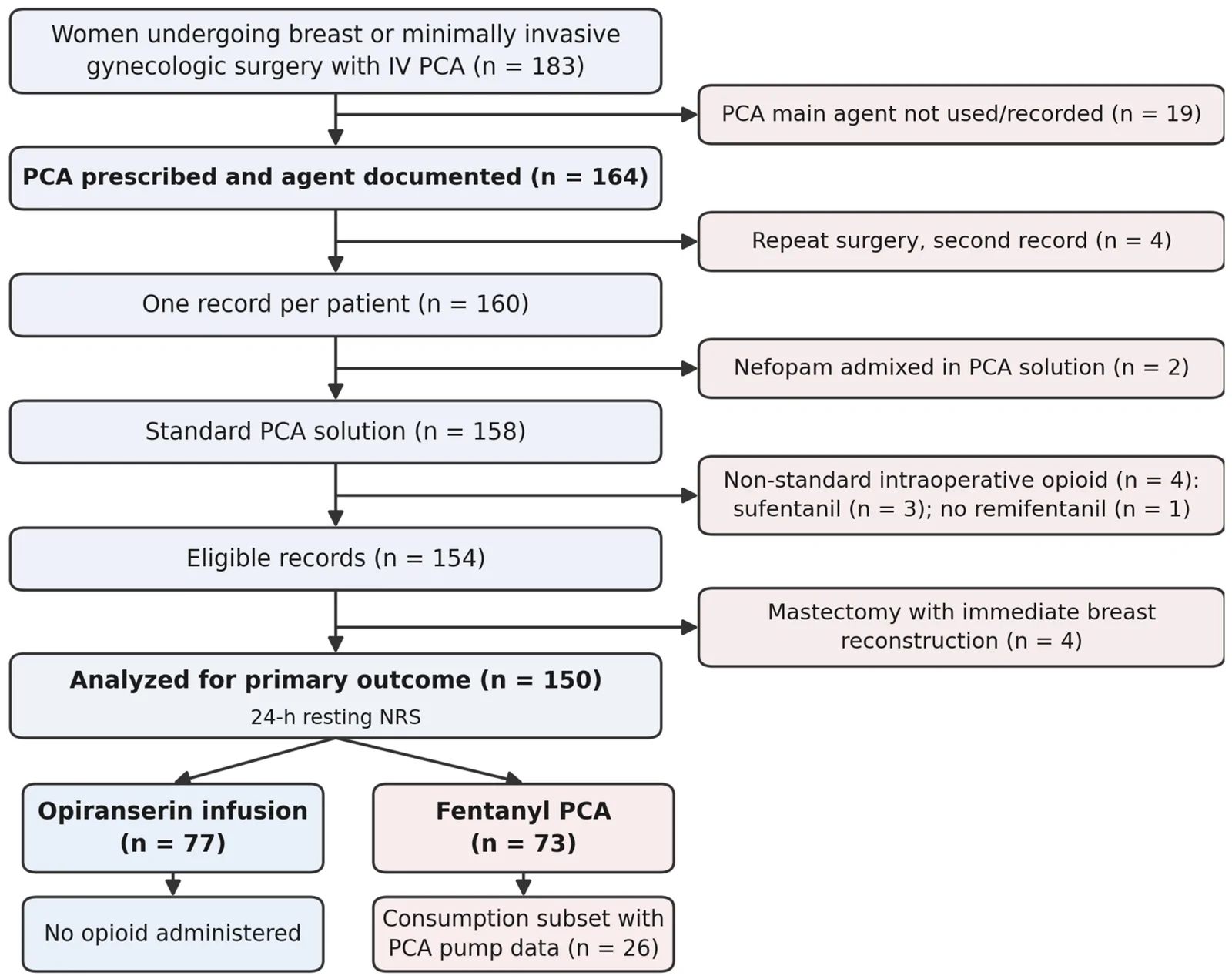
Study flow diagram.

Of 183 women screened, 19 were excluded because a PCA main agent was not used or documented, four were excluded because they were the second record of a repeat surgery, two were excluded because nefopam had been admixed in the PCA solution, and four were excluded because of a non-standard intraoperative opioid regimen (sufentanil in three; intraoperative fentanyl without remifentanil in one), leaving 154 records. A further four patients were excluded because their nipple-sparing mastectomies had been scheduled as combined procedures with the plastic surgery service; all four were in the fentanyl group, and their operations lasted 303 to 565 min, against 94 to 146 min for the nipple-sparing mastectomies retained in the cohort. The analysis cohort therefore comprised 150 patients—77 receiving opiranserin and 73 receiving fentanyl (Figure 1). No sampling, frequency matching, or group balancing was performed at any stage. Height, weight, and 24 h NRS were available for all patients, so all 150 contributed to every analysis. Results for the full 154-record cohort, and for a cohort from which every mastectomy was excluded, are reported as sensitivity analyses.

The groups were comparable in age, height, ASA physical status, and comorbidities, and all received volatile maintenance without total intravenous anesthesia or regional block (Table 1). Breast procedures comprised breast-conserving operations—breast-conserving surgery, wide excision, or mass excision—in 36 patients (30 opiranserin, six fentanyl) and mastectomy in eight patients, all of whom received fentanyl PCA. Consistent with the allocation process, however, patients receiving opiranserin underwent shorter operations (median, 55 min; IQR, 40–87 vs. 65 min; IQR, 52–94; *p* = 0.033; SMD, −0.34), received less intraoperative remifentanil (median, 450 µg; IQR, 350–600 vs. 550 µg; IQR, 450–800; *p* = 0.001; SMD, −0.58), more often underwent breast surgery (39.0% vs. 19.2%; *p* = 0.012) and less often underwent total laparoscopic hysterectomy (9.1% vs. 28.8%; *p* = 0.003); body mass index was slightly higher (median, 24.6 kg/m^2^; IQR, 21.5–27.5 vs. 23.0 kg/m^2^; IQR, 20.8–25.2; *p* = 0.027; SMD +0.27).

Antiemetic prophylaxis differed in composition, with dexamethasone used more often in the opiranserin group (39.0% vs. 2.7%) and palonosetron more often in the fentanyl group (40.3% vs. 61.6%). The proportion of patients receiving at least one intraoperative non-opioid analgesic was similar (84.4% vs. 89.0%, *p* = 0.476), although the mean number of drug classes used was lower in the opiranserin group (0.86 ± 0.39 vs. 1.11 ± 0.57, *p* = 0.002; a count variable, for which the mean is reported because the medians were identical), reflecting the pairing of opiranserin with nefopam as a standing combination against the more varied, physician-dependent choices accompanying fentanyl PCA; nefopam therefore predominated with opiranserin (74.0% vs. 46.6%, *p* = 0.001), whereas acetaminophen-containing agents were more frequent with fentanyl (acetaminophen, 2.6% vs. 38.4%; acetaminophen–ibuprofen, 9.1% vs. 26.0%).

### 3.2. Primary Outcome: Resting Pain at 24 h

**Figure 2:**
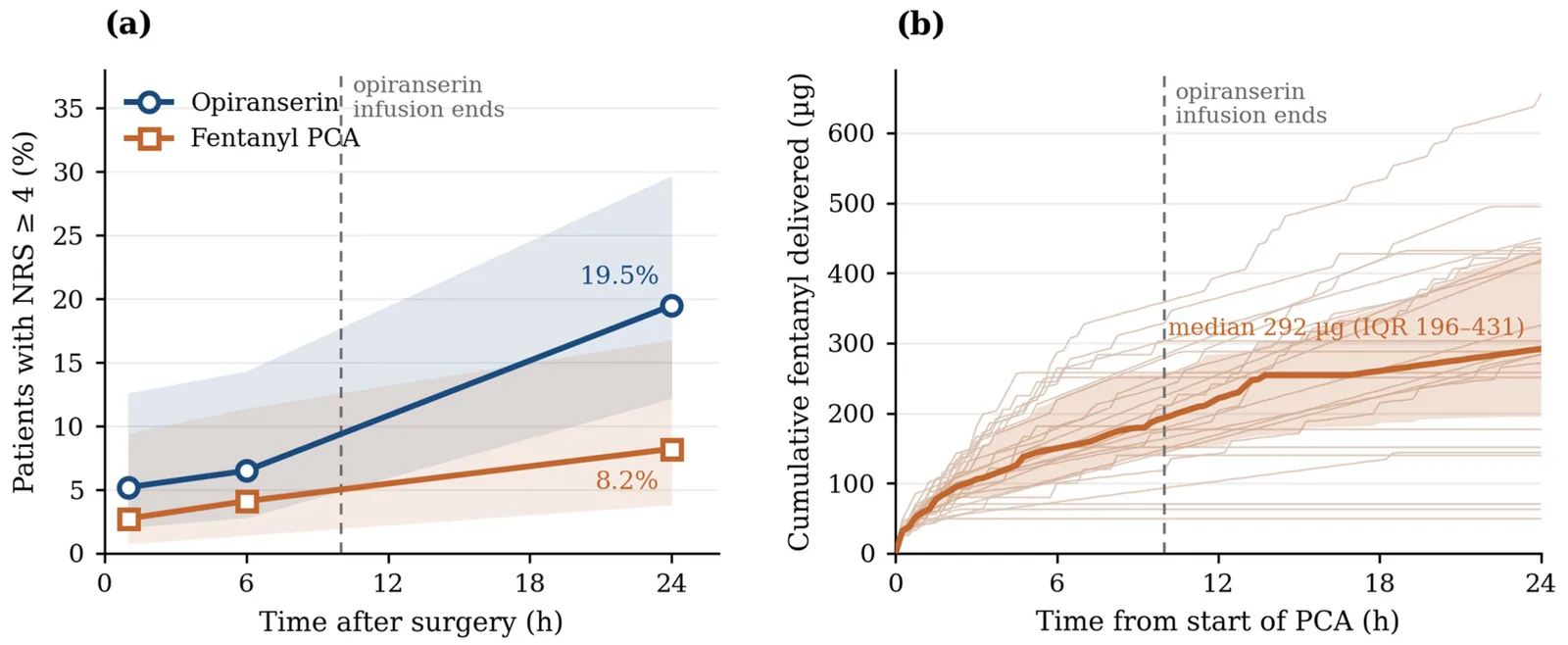
Postoperative time course over the first 24 h. (**a**) Proportion of patients reporting a resting numeric rating scale of 4 or more, the conventional threshold for moderate pain, in the opiranserin (*n* = 77) and fentanyl (*n* = 73) groups; shaded bands are 95% Wilson confidence intervals. (**b**) Cumulative fentanyl delivered in the subset of the fentanyl group with retrievable infusion-pump records (*n* = 26); faint lines denote individual patients, the solid line the median, and the shaded band the interquartile range. In both panels, the dashed line marks the 10 h fixed duration of the opiranserin infusion, which began before skin incision and therefore ended approximately 9 h after the end of surgery in a typical case. NRS: numeric rating scale, PCA: patient-controlled analgesia.

No statistically significant between-group difference in resting NRS was detected while the opiranserin infusion was running. Median scores were 2.0 (IQR 2.0–2.0) in both groups on arrival in the PACU (*p* = 0.965) and 1.0 (1.0–2.0) in both groups at 6 h (*p* = 0.675). The larger difference at the 24 h assessment, by which time the infusion had been complete for approximately 15 h, was an observed exploratory pattern rather than a demonstrated divergence (Figure 2a).

**Figure 3:**
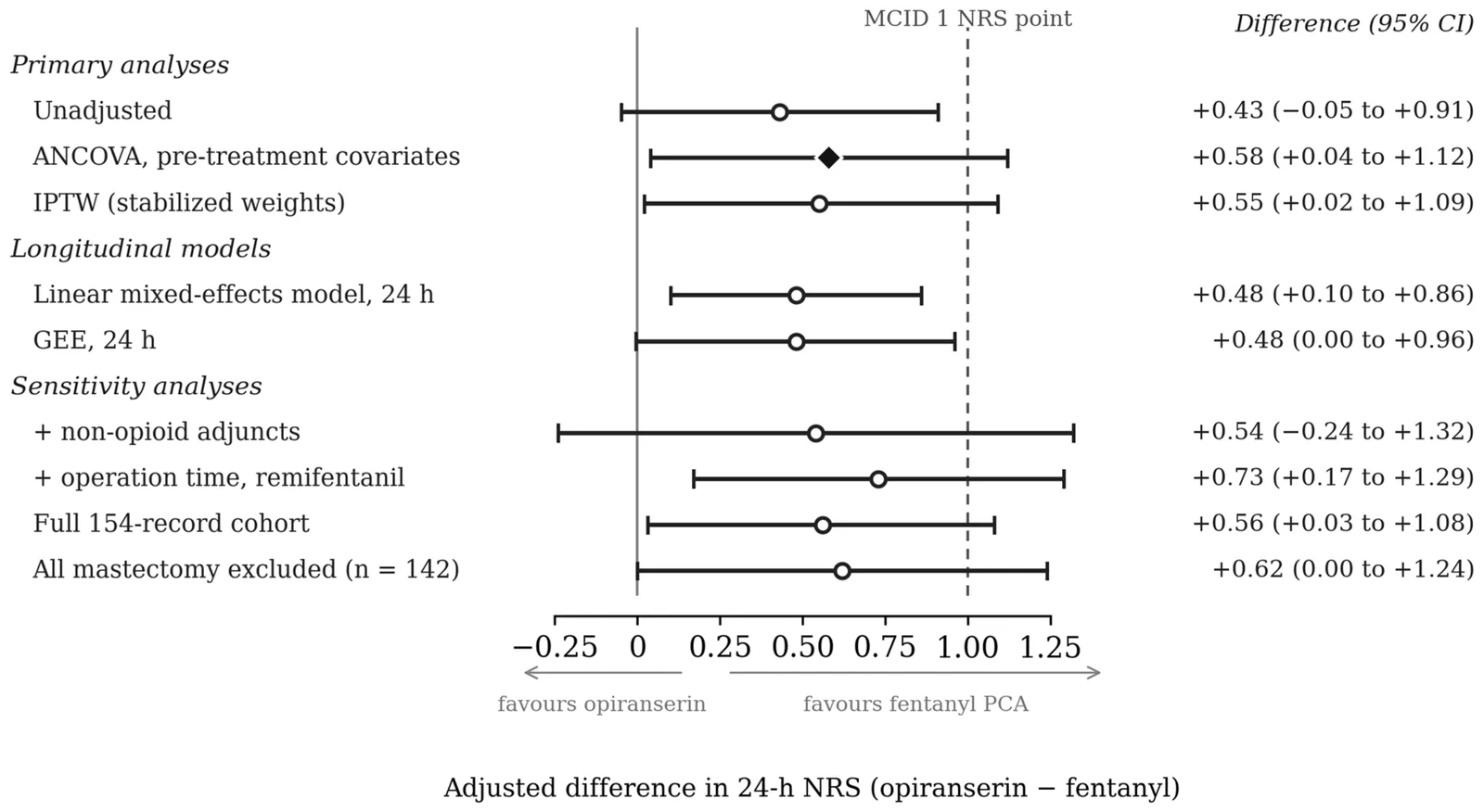
Between-group difference in 24 h numeric rating scale across analytic models. Markers are adjusted differences with 95% confidence intervals; the filled diamond denotes the primary analysis. The solid vertical line marks no difference and the dashed line the minimal clinically important difference of 1 NRS point. A positive value indicates higher pain with opiranserin. The proportional odds ordinal model is reported in Table 3 only because its estimate is an odds ratio and is not on the same scale. IPTW: inverse-probability-of-treatment weighting, ANCOVA: analysis of covariance, GEE: generalized estimating equations, NRS: numeric rating scale.

At 24 h, the primary outcome, median resting NRS, was 1.0 (IQR 1.0–2.0) in the opiranserin group and 1.0 (1.0–1.0) in the fentanyl group; the unadjusted comparison was not significant (*p* = 0.249; Table 2). Corresponding means were 1.9 and 1.4 points, reported here because the adjusted analyses below estimate a difference in means. After adjustment for pre-treatment covariates, the difference was 0.58 points higher with opiranserin (95% CI, 0.04 to 1.12; *p* = 0.036), and inverse-probability-of-treatment weighting gave a closely similar estimate of 0.55 (0.02 to 1.09; *p* = 0.042), confirmed by bootstrap resampling (0.56, −0.01 to 1.12). The point estimates were smaller than the MCID of 1 point, but the upper confidence limits approach or exceed it so that a clinically relevant increase in pain cannot be excluded (Table 3, Figure 3). Estimates were consistent in direction across all models, from +0.43 unadjusted to +0.73 when operation time and remifentanil were added and +0.54 (95% CI, −0.24 to +1.32; *p* = 0.177) when concomitant non-opioid analgesics were added; they were also stable across cohort definitions, at +0.56 (0.03 to 1.08) in the full 154-record cohort and +0.62 (0.00 to 1.24) when every mastectomy was excluded (Table 3). Moderate or severe pain, defined as an NRS of 4 or more at 24 h, was recorded in 15 patients (19.5%) in the opiranserin group and six (8.2%) in the fentanyl group, a risk difference of 11.3 percentage points (95% CI, −0.0 to +22.4) and an adjusted odds ratio of 3.76 (95% CI, 1.34 to 11.82; *p* = 0.011).

A linear mixed-effects model with a random patient intercept and a generalized estimating equation with an exchangeable working correlation, each fitted to all three time points with a group-by-time interaction, gave concordant estimates. By the mixed model, the difference was +0.13 (95% CI, −0.25 to +0.51) at the PACU, +0.18 (−0.20 to +0.56) at 6 h, and +0.48 (+0.10 to +0.86) at 24 h; by the generalized estimating equation, it was +0.13 (−0.14 to +0.40), +0.18 (−0.18 to +0.55), and +0.48 (−0.00 to +0.96). The intraclass correlation was 0.187. The group-by-time interaction was not statistically significant (likelihood ratio test, 2.53 on 2 degrees of freedom; *p* = 0.282), so the apparent widening of the between-group difference across time points is reported as an observed pattern rather than as a demonstrated divergence, and the study was not powered to detect an interaction of this size. The proportional odds ordinal model gave an odds ratio of 1.74 (95% CI, 0.82 to 3.70; *p* = 0.150) for a higher 24 h NRS category with opiranserin.

After weighting, stabilized weights ranged from 0.53 to 3.60 (median, 0.83; interquartile range, 0.71 to 1.10) and the effective sample size was 119.9 of 150 (80%). Propensity scores spanned 0.107 to 0.842 in the opiranserin group and 0.089 to 0.889 in the fentanyl group; the region of common support was 0.107 to 0.842; three fentanyl-group patients (4.1%) lay marginally outside this region and were retained in the weighted analysis. Absolute standardized mean differences fell from a maximum of 0.52 before weighting to 0.122 after weighting; among the covariates included in the propensity score model, the only covariates remaining above the prespecified threshold of 0.10 were hypertension (0.111) and diabetes mellitus (0.122), with 24 and 11 events, respectively (Figure S1).

### 3.3. Postoperative Nausea, Vomiting, and Rescue Medication

PONV occurred in 9.1% of the opiranserin group and 11.0% of the fentanyl group (risk difference, −1.9 percentage points; 95% CI, −12.2 to +8.2; adjusted odds ratio, 0.87, 0.26 to 2.81), and rescue antiemetic use was 2.6% and 2.7%, respectively. Prophylactic regimens differed in composition, and two or more antiemetics were given more often in the opiranserin group (14.3% vs. 1.4%, *p* = 0.005). Because only 15 PONV events occurred in the whole cohort, models were fitted by Firth penalized logistic regression. The odds ratio for PONV with opiranserin was 0.82 (95% CI, 0.28 to 2.33) unadjusted, 0.83 (0.24 to 2.60) after adjustment for dexamethasone, and 0.75 (0.22 to 2.37) after adjustment for all prophylactic antiemetics, with events per variable falling from 15.0 to 3.8 across these models; models additionally including nefopam and demographic covariates are given in Table S1 and have events per variable of 3.0 and 2.1. In every model, the confidence interval was wide so that the data are compatible with anything from a substantial reduction to a more than twofold increase in PONV. Ramosetron was admixed in the opiranserin infusate from January 2026 onward; PONV did not differ between patients who received the admixture and those who did not (9.6% vs. 8.0%, *p* = 1.000). Early discontinuation of the analgesic device, and the reason recorded for it, were verified against the nursing and physician records of every patient flagged as having discontinued. In three fentanyl-group patients, the flag was not corroborated by any entry in the record and was reclassified as a data-entry error; verified early discontinuation therefore occurred in eight of 77 opiranserin-group patients (10.4%) and seven of 73 fentanyl-group patients (9.6%; risk difference, +0.8 percentage points; 95% CI, −9.4 to +10.9). Discontinuation was attributed to an adverse event in eight opiranserin-group (10.4%) and six fentanyl-group patients (8.2%); the remaining fentanyl-group discontinuation followed removal of the intravenous cannula for infusion-site discomfort. In the opiranserin group, the recorded events were nausea in five patients, dizziness in two, and vomiting, headache, diaphoresis, hypotension, and facial hypoesthesia in one each; in the fentanyl group, they were nausea in six, dizziness in two, and chest discomfort in one. A rescue antiemetic was administered at discontinuation to one patient in each group (ondansetron, 4 mg, and ramosetron, 0.3 mg), and the infusion was subsequently restarted in one opiranserin-group and two fentanyl-group patients. Events were graded as mild in five, moderate in two, and severe in one opiranserin-group patient and mild in all fentanyl-group patients. The median interval from the start of the analgesic infusion to discontinuation was 6.9 h (interquartile range, 3.6–8.4) in the opiranserin group and 10.7 h (5.8–13.5) in the fentanyl group (*p* = 0.181). The single severe event was hypotension (62/33 mmHg with a heart rate of 46 beats·min^−1^) recorded 8 h 22 min after the start of an opiranserin infusion following total laparoscopic hysterectomy; it resolved with intravenous fluid and leg elevation, without vasopressor support, intensive care admission, or prolongation of hospital stay and, therefore, did not meet criteria for a serious adverse event. Causality was not formally adjudicated, and the event is reported as documented rather than as attributed. No event in either group met criteria for a serious adverse event. Among patients who developed PONV, five of seven (71%) in the opiranserin group and five of eight (63%) in the fentanyl group had their analgesic device discontinued early. Any rescue analgesic was given to 26.0% versus 17.8% of patients (risk difference, +8.2 percentage points; 95% CI, −5.2 to +21.1; adjusted odds ratio, 2.17, 0.95 to 5.15), mostly ketorolac (23.4% vs. 15.1%); the timing of these administrations was not recorded. The mean number of rescue analgesic doses per patient was identical in the two groups (0.26 vs. 0.26), and two rescue antiemetic doses were given in each group. Rescue opioid was given to one patient in the fentanyl group and to none in the opiranserin group. Although rescue opioids were available on the ward to either group, no patient managed with opiranserin required one so that this group received no opioid at any point after surgery, either through the analgesic infusion or as rescue medication.

### 3.4. Fentanyl Consumption in the Comparator Group

Infusion-pump event logs were electronically retrievable for 26 patients in the fentanyl group, representing 36% of that group; logs were available for 14 of 15 patients operated on in November 2025, for 12 of 17 operated on in December 2025, and for none of the 41 operated between January and April 2026. In this subset, cumulative fentanyl delivered within 24 h was 306 ± 151 µg, of which 216 ± 92 µg was given during the first 12 h and a further 90 ± 86 µg between 12 and 24 h (Figure 2b). Individual 24 h consumption ranged from 50 to 656 µg, and 103 of 308 bolus attempts (33%) were not delivered because of the lockout interval; the mean of the per-patient delivered-to-attempted ratios was 0.86 ± 0.25, calculated in the 25 patients who made at least one demand. Patients with and without retrievable logs did not differ in age (SMD, −0.01), body mass index (SMD, −0.05), operation time (SMD, −0.09), intraoperative remifentanil (SMD, +0.12), 24 h NRS, PONV (15.4% vs. 8.5%), or early discontinuation (11.5% vs. 8.5%); rescue analgesic use was more frequent among those with retrievable logs (34.6% vs. 8.5%, *p* = 0.009; Table S2). Log availability was determined by the date of surgery relative to the interface failure rather than by any patient characteristic; this single difference cannot, however, be excluded as a source of selection bias. These data are descriptive and are not necessarily representative of the whole group.

## 4. Discussion

Opiranserin has previously been evaluated only as an adjunct to opioid patient-controlled analgesia (PCA); in this cohort it replaced the opioid entirely, to our knowledge for the first time. In women undergoing breast or minimally invasive gynecologic surgery, a programmed multistep infusion with the demand bolus function disabled and no opioid in the reservoir served as the sole analgesic base of the postoperative regimen. Postoperative opioid exposure was therefore eliminated rather than reduced. In routine practice, the regimen was delivered as planned, and resting pain differed from that with fentanyl PCA by less than 1 NRS point on average. A larger minority of opiranserin-treated patients nevertheless reported moderate or severe pain at 24 h. Even so, where postoperative opioid exposure is to be avoided altogether, these observations suggest that this regimen is one practical option and not merely a theoretical possibility. What they show is that the regimen can be delivered, not that it matches fentanyl PCA for analgesia. The sample size was calculated to detect a one-point difference within a conventional superiority framework, and no non-inferiority margin was set in advance, so neither equivalence nor non-inferiority can be read from these data, and the regimen should, for now, be used with caution and where avoiding postoperative opioid exposure is the explicit aim.

Opioid-sparing regimens built on other non-opioid agents have been reported. In pelviscopic gynecologic surgery, a continuous ketorolac infusion combined with patient-controlled fentanyl reduced opioid-related side effects while maintaining analgesia [22], and after total knee arthroplasty an opioid-sparing PCA containing ketorolac and nefopam was non-inferior to a fentanyl-based regimen with fewer adverse events [23]. Both, however, retained a patient-controlled opioid bolus for breakthrough pain, so that opioid exposure was reduced but not removed. The present protocol dispensed with that option altogether, and did so under conditions that were, if anything, unfavourable: the infusion was fixed at 10 h and had been complete for roughly 15 h by the time of the primary assessment, whereas fentanyl PCA remained connected throughout. While the infusion was running no significant between-group difference was detected: resting scores differed by less than 0.2 points at both the PACU and 6 h assessments (*p* = 0.965 and 0.675). The larger difference appeared at 24 h, after coverage had ended, and the adjusted estimate of 0.58 points was smaller than the MCID of 1 point, although the confidence interval does not exclude a clinically relevant increase in pain. Because the group-by-time interaction was not statistically significant, the apparent widening across time points is described here as a pattern observed in exploratory analysis rather than as a demonstrated divergence. With the infusion duration fixed at 10 h in every patient, the 24 h difference cannot be separated into a shortfall of the agent and a shortfall of coverage.

The infusion could not be extended. Opiranserin is approved in Korea for the short-term treatment of moderate to severe acute postoperative pain as a single fixed course: the dosage is 160 mg over 30 min followed by 840 mg over 9.5 h for a total of 1000 mg in 500 mL over 10 h. Repeat administration falls outside that approval, so a second bag was not available even to patients who asked for one on the ward after the infusion had finished. Cost argues in the same direction, since the regimen is not reimbursed and is paid for out of pocket, but it was the approval that settled the matter. Coverage was therefore fixed at 10 h in every patient, and the interval between the end of the infusion and the 24 h assessment follows from the approved regimen rather than from the design of this study.

A regimen that removes the opioid invites the objection that other analgesics must have been substituted for it. The present data do not support that explanation. Nefopam predominated with opiranserin (74.0% vs. 46.6%) and acetaminophen-containing agents with fentanyl (2.6% vs. 38.4% for acetaminophen), but the number of adjunct drug classes was in fact lower in the opiranserin group (0.86 vs. 1.11, *p* = 0.002). Similar pain scores were thus achieved with fewer classes of adjunct, not more, and nefopam was given to nearly half the fentanyl group as well, so that the two regimens differed principally in the content of the PCA reservoir. Nefopam alone, moreover, is not sufficient to account for the result: we previously reported that nefopam as the sole agent within a PCA failed to demonstrate non-inferiority to fentanyl after laparoscopic gynecologic surgery, the upper confidence limit exceeding a margin of 1 NRS point [8]. Taken together, these observations are consistent with opiranserin contributing meaningfully to the regimen, although its independent analgesic effect cannot be established from these data; what is compared here is an opiranserin-based multimodal regimen against a fentanyl-based multimodal regimen, and the 24 h difference was no longer significant once the concomitant non-opioid drugs were entered into the model (0.54 points; 95% CI, −0.24 to 1.32). A second objection is that the opioid-free status of that group might reflect restricted prescribing rather than the regimen itself. Rescue opioids were prescribed at the ward’s discretion and were used in the fentanyl group, yet no patient managed with opiranserin required one; the absence of rescue opioid use is not by itself evidence that analgesia was adequate in every patient, but it does indicate that the opioid-free status of that group did not result from restricted prescribing.

The absence of any reduction in PONV is notable, since this was the clinical problem that prompted the protocol. Among patients who developed PONV, five of seven in the opiranserin group and five of eight in the fentanyl group had their analgesic device discontinued early, yet removing the opioid lowered neither the incidence of PONV (9.1% vs. 11.0%) nor early discontinuation (10.4% vs. 9.6%). Two explanations are plausible. First, nausea and vomiting are themselves among the most frequently reported adverse events of opiranserin [9,10,11,12], so the benefit of eliminating opioid-induced nausea may have been offset by drug-related nausea; agents substituted for an opioid carry adverse-effect profiles of their own. Second, absolute event rates were low in both groups despite a uniformly high-risk population, possibly in the context of the routine antiemetic prophylaxis that both groups received [14]. Ramosetron was admixed in the opiranserin infusate from January 2026 onward, but PONV did not differ between patients who received the admixture and those who did not (9.6% vs. 8.0%, *p* = 1.000), and the odds ratio for PONV changed little after adjustment for dexamethasone, for all prophylactic antiemetics, or for nefopam. A comparable pattern has been reported after laparoscopic gynecologic surgery, where replacing fentanyl with nefopam in the PCA did not lower PONV under prophylactic ramosetron [8], and the most recent consensus guidance notes that although opioid-free anesthesia reduces PONV compared with opioid-based anesthesia, it is unclear whether the same holds against multimodal opioid-sparing analgesia [24]. With PONV in only 15 of the 150 patients, the confidence intervals were wide enough to encompass both a substantial reduction and a substantial increase, and differences in antiemetic prophylaxis and in the adverse-effect profiles of the drugs used may also have contributed. Where effective pharmacological prophylaxis is already in place, removing a single Apfel risk factor may yield little additional benefit, a consideration relevant to any institution contemplating an opioid-free protocol primarily for its antiemetic advantage.

Several limitations qualify these findings. Allocation was not random: in addition to patient consent, the anesthesiology and surgical teams jointly elected conventional fentanyl PCA when a larger or more painful operation was anticipated, which produced shorter operations, lower remifentanil doses, and fewer total laparoscopic hysterectomies in the opiranserin group. Confounding by indication, willingness to pay for the more expensive regimen, anticipated pain severity, and unmeasured socioeconomic or clinician-level factors therefore all bear on the comparison. Payment itself may have selected for patients who differ in ways we did not record: means can track with educational and cultural background, with what patients expect of postoperative pain relief, and with how reliably they take oral rescue analgesia, and each of these could shape reported pain independently of the drug. This selection would be expected to favor opiranserin, yet 24 h pain scores were slightly higher in that group. Measured imbalances were addressed by covariance adjustment and weighting, but unmeasured determinants of that clinical judgement cannot be controlled, and patients knew that they were receiving a non-opioid regimen, so an expectation effect cannot be excluded. Concomitant analgesia was not standardized, and although the adjunct burden was lower with opiranserin, the 24 h difference was no longer significant once these drugs were entered into the model (*p* = 0.177); what these data support is therefore the feasibility of an opioid-free multimodal protocol in this population rather than the analgesic potency of opiranserin as a single agent. The fixed infusion also cannot be titrated: moderate or severe pain at 24 h was recorded in 19.5% versus 8.2% of patients, an adjusted odds ratio of 3.76 (95% CI, 1.34 to 11.82), while individual requirement in the comparator group ranged from 50 to 656 µg of fentanyl over 24 h [19], so that a subset was inadequately covered by a dose that is identical for everyone. Because this was a secondary outcome analyzed without adjustment for multiplicity, the finding is hypothesis-generating. Safety information was limited by the retrospective design. No standardized adverse event case report form was used; events were abstracted from routine clinical records after the fact, and causality was not formally adjudicated. The figures reported here therefore describe the events documented at the time of discontinuation and will understate milder events that did not prompt a change in management, so they should be read as a safety signal rather than as a full characterization of the adverse event profile. The cohort was also defined more broadly than the approved protocol anticipated in that breast operations more extensive than breast-conserving surgery were included; estimates were, however, stable when these were excluded. All patients were women, all were non-smokers, and none had a documented history of PONV or motion sickness so that external validity to men, to smokers, to patients at different baseline PONV risk, and to other surgical populations is untested. Finally, the sample size was calculated for a 1-point difference in 24 h NRS and not for PONV, within a conventional superiority framework that cannot establish non-inferiority; no equivalence margin was prespecified; opioid consumption could be retrieved for only 26 fentanyl-group patients; pain was recorded at rest at three routine time points [17]; and secondary analyses were exploratory and unadjusted for multiplicity. A prospective trial with standardized adjuncts, an assessment point inside the coverage window, a regimen whose duration or dose can be adapted to individual requirement, and a prespecified non-inferiority margin are required.

## 5. Conclusions

A postoperative opioid-free regimen combining opiranserin with non-opioid adjuncts was feasible after breast and minimally invasive gynecologic surgery. Resting pain differed from that with fentanyl PCA by less than 1 NRS point on average, even though the infusion ended at 10 h while the comparator remained connected throughout, although the confidence intervals do not exclude a clinically relevant increase in pain, and a larger minority of patients receiving opiranserin reported moderate or severe pain at 24 h. Postoperative opioid exposure was eliminated rather than reduced: rescue opioids remained available and were used in the comparator group, but no patient receiving opiranserin required one, removing the exposure that underlies persistent postoperative opioid use in opioid-naive patients [25] and the opioid-related adverse events that prolong hospital stay and increase cost [26]. The anticipated reduction in PONV was not, however, observed, possibly in the context of low event rates and routine antiemetic prophylaxis in both groups. These data show feasibility, not comparative efficacy: this was not a non-inferiority trial, and the regimen is best reserved for patients in whom avoiding postoperative opioid exposure is itself the aim. Whether opiranserin can carry this role unaided remains to be established in a prospective controlled trial.

## Figures and Tables

**Table 1:** Baseline and perioperative characteristics.

Variable	Opiranserin (*n* = 77)	Fentanyl (*n* = 73)	SMD	*p*
*Demographics*				
Age (yr)	46.0 (39.0–54.0)	46.0 (34.0–51.0)	+0.05	0.604
Height (cm)	159.4 (156.5–163.4)	159.4 (156.3–162.5)	+0.09	0.554
Weight (kg)	61.8 (55.0–69.8)	57.3 (50.0–65.3)	+0.28	0.027
Body mass index (kg/m^2^)	24.6 (21.5–27.5)	23.0 (20.8–25.2)	+0.27	0.027
ASA physical status 1, *n* (%)	1 (1.3)	0 (0.0)		1.000
ASA physical status 2, *n* (%)	73 (94.8)	68 (93.2)		0.741
ASA physical status 3, *n* (%)	3 (3.9)	5 (6.8)		0.486
*Comorbidities, *n* (%)*				
Hypertension	13 (16.9)	11 (15.1)		0.826
Diabetes mellitus	6 (7.8)	5 (6.8)		1.000
Thyroid disease	3 (3.9)	5 (6.8)		0.486
*Surgery and anesthesia*				
Breast surgery, *n* (%)	30 (39.0)	14 (19.2)		0.012
Pelviscopic surgery, *n* (%)	28 (36.4)	20 (27.4)		0.294
Robot-assisted surgery, *n* (%)	12 (15.6)	18 (24.7)		0.220
Total laparoscopic hysterectomy, *n* (%)	7 (9.1)	21 (28.8)		0.003
Operation time (min)	55 (40–87)	65 (52–94)	−0.34	0.033
Desflurane, *n* (%)	49 (63.6)	39 (53.4)		0.246
Sevoflurane, *n* (%)	28 (36.4)	34 (46.6)		0.246
Intraoperative remifentanil (µg)	450 (350–600)	550 (450–800)	−0.58	0.001
*Antiemetic prophylaxis, *n* (%)*				
Palonosetron	31 (40.3)	45 (61.6)		0.010
Ramosetron	23 (29.9)	25 (34.2)		0.602
Dexamethasone	30 (39.0)	2 (2.7)		<0.001
Two or more agents	11 (14.3)	1 (1.4)		0.005
*Intraoperative non-opioid analgesics*				
Any agent, *n* (%)	65 (84.4)	65 (89.0)		0.476
Number of drug classes ^a^	0.86 ± 0.39	1.11 ± 0.57	−0.52	0.002
Nefopam, *n* (%)	57 (74.0)	34 (46.6)		0.001
Acetaminophen, *n* (%)	2 (2.6)	28 (38.4)		<0.001
Acetaminophen–ibuprofen, *n* (%)	7 (9.1)	19 (26.0)		0.009

Values are presented as median (interquartile range) or number (%); every continuous variable departed from normality in at least one group on the Shapiro–Wilk test, and groups were compared with the Mann–Whitney U test. ^a^ Mean ± SD is shown for this count variable because the medians were identical in the two groups. SMD: standardized mean difference, ASA: American Society of Anesthesiologists. *p* values were derived from the Mann–Whitney U test for continuous variables and the Fisher exact test for categorical variables. All patients were female non-smokers without a documented history of postoperative nausea and vomiting or motion sickness, and all received volatile maintenance; none received total intravenous anesthesia or a regional block. Individual antiemetics and non-opioid analgesics are counted by any use and may overlap. Antiemetic prophylaxis and intraoperative non-opioid analgesics were prescribed at the discretion of the attending team and were not standardized between groups.

**Table 2:** Postoperative outcomes.

Outcome	Opiranserin (*n* = 77)	Fentanyl (*n* = 73)	Risk Difference, % (95% CI)	Adjusted OR (95% CI)	*p*
*Resting pain (NRS 0–10)*					
PACU	2.0 (2.0–2.0)	2.0 (2.0–2.0)			0.965
6 h	1.0 (1.0–2.0)	1.0 (1.0–2.0)			0.675
24 h (primary outcome)	1.0 (1.0–2.0)	1.0 (1.0–1.0)			0.249
NRS ≥ 4 at 24 h, *n* (%)	15 (19.5)	6 (8.2)	+11.3 (−0.0 to +22.4)	3.76 (1.34–11.82)	0.011
*Postoperative nausea and vomiting*					
PONV, *n* (%)	7 (9.1)	8 (11.0)	−1.9 (−12.2 to +8.2)	0.87 (0.26–2.81)	0.811
Any rescue antiemetic, *n* (%)	2 (2.6)	2 (2.7)			1.000
*Rescue analgesia*					
Any rescue analgesic, *n* (%)	20 (26.0)	13 (17.8)	+8.2 (−5.2 to +21.1)	2.17 (0.95–5.15)	0.066
Ketorolac, *n* (%)	18 (23.4)	11 (15.1)			0.220
Acetaminophen, *n* (%)	2 (2.6)	2 (2.7)			1.000
Rescue opioid, *n* (%)	0 (0.0)	1 (1.4)			0.487
*Patient-controlled analgesia*					
Discontinued early, *n* (%)	8 (10.4)	7 (9.6)	+0.8 (−9.4 to +10.9)	0.82 (0.21–3.06)	0.768
Due to an adverse effect, *n* (%)	8 (10.4)	6 (8.2)			0.781

Values are presented as median (interquartile range) or number (%). NRS: numeric rating scale (0–10, at rest), PACU: post-anesthesia care unit, PONV: postoperative nausea and vomiting. Risk differences are reported with Newcombe hybrid score confidence intervals. Adjusted odds ratios were estimated by Firth penalized logistic regression with profile penalized-likelihood confidence intervals, adjusting for age, body mass index, ASA physical status, comorbidities, and procedure type; penalization was used because of the small number of events. Rescue analgesic subcategories are counted by drug class and may overlap when more than one class was administered. Rescue opioids were available on the ward to either group; no patient managed with opiranserin required one, so that group received no opioid at any point after surgery. Early discontinuation and the reason recorded for it were verified against the nursing and physician records of every patient flagged as having discontinued; in three fentanyl-group patients, no corresponding entry was found and the flag was reclassified as a data-entry error. *p* values are from the Fisher exact test, except in rows reporting an adjusted odds ratio, where the *p* value is that of the corresponding Firth penalized model; the unadjusted Fisher exact *p* value for NRS ≥ 4 at 24 h was 0.060. Secondary analyses were exploratory and were not adjusted for multiplicity.

**Table 3:** Between-group difference in 24 h numeric rating scale across analytic models.

Analytic Model	Adjusted Difference in 24 h NRS (95% CI)	*p*
Unadjusted	+0.43 (−0.05 to +0.91)	0.076
ANCOVA, pre-treatment covariates (primary)	+0.58 (+0.04 to +1.12)	0.036
IPTW (stabilized weights)	+0.55 (+0.02 to +1.09)	0.042
IPTW, bootstrap (2000 replicates)	+0.56 (−0.01 to +1.12)	—
Linear mixed-effects model, 24 h contrast	+0.48 (+0.10 to +0.86)	0.013
GEE, 24 h contrast	+0.48 (−0.00 to +0.96)	0.050
Proportional odds ordinal model, odds ratio	1.74 (0.82 to 3.70)	0.150
Sensitivity: + non-opioid adjuncts	+0.54 (−0.24 to +1.32)	0.177
Sensitivity: + operation time, remifentanil	+0.73 (+0.17 to +1.29)	0.011
Sensitivity: full 154-record cohort	+0.56 (+0.03 to +1.08)	0.037
Sensitivity: all mastectomy excluded (*n* = 142)	+0.62 (+0.00 to +1.24)	0.050

Adjusted difference = opiranserin minus fentanyl; a positive value indicates higher pain with opiranserin. Complete-case analysis, *n* = 150. The primary model adjusts for pre-treatment covariates only: age, body mass index, ASA physical status, hypertension, diabetes mellitus, thyroid disease, and planned procedure type. Operation time and intraoperative remifentanil dose are determined after treatment allocation, since opiranserin was started before skin incision, and are therefore treated as potential mediators rather than baseline confounders; a model including them is shown as a sensitivity analysis. An ordinal odds ratio above 1 indicates higher odds of a higher NRS category with opiranserin. GEE: generalized estimating equations with an exchangeable working correlation. IPTW: inverse-probability-of-treatment weighting, using stabilized weights truncated at the 99th percentile. Heteroscedasticity-consistent (HC3) robust standard errors were applied to all models, and IPTW estimates were verified by bootstrap resampling with 2000 replicates. No equivalence or non-inferiority margin was prespecified; comparison against the minimal clinically important difference of 1 point is descriptive.

## Data Availability

The data presented in this study are available on request from the corresponding authors due to privacy restrictions applying to identifiable clinical records.

## Supplementary Materials

The following supporting information can be downloaded at: https://www.mdpi.com/article/10.3390/jcm15208062/s1. Figure S1: Covariate balance before and after inverse-probability-of-treatment weighting; Table S1: Firth penalized logistic regression models for postoperative nausea and vomiting; Table S2: Characteristics of fentanyl-group patients with and without retrievable infusion-pump logs.
