# Supplementary material for: Postoperative Opioid-Free Multistep Opiranserin Infusion Versus Fentanyl Patient-Controlled Analgesia After Breast or Minimally Invasive Gynecologic Surgery: A Retrospective Cohort Study
Source: J Clin Med. 2026 Oct 17;15(20):8062. doi: 10.3390/jcm15208062 (PMC13706320; doi:10.3390/jcm15208062)
Supplement: Supplementary file 1 [file jcm-15-08062-s001.zip › jcm-4565152-supplementary.pdf]

# Supplementary Materials

*Postoperative Opioid-Free Multistep Opiranserin Infusion Versus Fentanyl Patient-Controlled Analgesia After Breast or Minimally Invasive Gynecologic Surgery: A Retrospective Cohort Study*

**Table S1.** Firth penalized logistic regression models for postoperative nausea and vomiting.

| Model and covariate | Odds ratio | 95% CI | p |
|---|---|---|---|
| *Model 1 (events 15; variables 1; EPV 15.0)* | | | |
| Opiranserin group | 0.82 | 0.28–2.33 | 0.707 |
| *Model 2 (events 15; variables 2; EPV 7.5)* | | | |
| Opiranserin group | 0.83 | 0.24–2.60 | 0.752 |
| Dexamethasone | 1.09 | 0.24–4.36 | 0.901 |
| *Model 3 (events 15; variables 4; EPV 3.8)* | | | |
| Opiranserin group | 0.75 | 0.22–2.37 | 0.625 |
| Dexamethasone | 0.52 | 0.08–2.97 | 0.474 |
| Palonosetron | 0.26 | 0.05–1.52 | 0.132 |
| Ramosetron | 0.37 | 0.06–2.15 | 0.265 |
| *Model 4 (events 15; variables 5; EPV 3.0)* | | | |
| Opiranserin group | 0.80 | 0.22–2.60 | 0.708 |
| Dexamethasone | 0.53 | 0.08–3.01 | 0.487 |
| Palonosetron | 0.27 | 0.05–1.61 | 0.147 |
| Ramosetron | 0.36 | 0.06–2.10 | 0.250 |
| Nefopam | 0.80 | 0.26–2.58 | 0.702 |
| *Model 5 (events 15; variables 7; EPV 2.1)* | | | |
| Opiranserin group | 0.84 | 0.23–2.91 | 0.789 |
| Dexamethasone | 0.57 | 0.08–3.15 | 0.533 |
| Palonosetron | 0.32 | 0.06–1.77 | 0.187 |
| Ramosetron | 0.43 | 0.07–2.49 | 0.347 |
| Nefopam | 0.72 | 0.23–2.33 | 0.576 |
| Age (per year) | 1.04 | 1.00–1.08 | 0.084 |
| BMI (per $kg/m^2$) | 1.01 | 0.87–1.14 | 0.924 |

Odds ratios are from Firth penalized logistic regression with profile penalized-likelihood confidence intervals. There were 15 events of postoperative nausea and vomiting in the cohort of 150 patients (7 of 77 with opiranserin, 8 of 73 with fentanyl). EPV: events per variable. Models 1 to 3 are reported in the main text; models 4 and 5 are provided for completeness and should be interpreted with caution given events per variable of 3.0 and 2.1. CI: confidence interval.

**Table S2.** Characteristics of fentanyl-group patients with and without retrievable infusion-pump logs.

| Variable | Logs retrievable (n = 26) | Logs not retrievable (n = 47) | SMD | p |
|---|---|---|---|---|
| Age (yr) | 46 (34–51) | 46 (36–51) | −0.01 | 0.904 |
| Height (cm) | 160.8 (155.5–162.5) | 158.7 (156.4–162.4) | +0.02 | 0.576 |
| Weight (kg) | 58.0 (51.1–64.4) | 57.2 (50.0–65.8) | −0.02 | 0.936 |
| Body mass index ($kg/m^2$) | 22.7 (21.0–24.6) | 23.2 (19.9–25.6) | −0.05 | 0.995 |
| Operation time (min) | 66 (56–88) | 64 (49–97) | −0.09 | 0.782 |
| Intraoperative remifentanil (μg) | 650 (462–975) | 510 (430–725) | +0.12 | 0.302 |
| PACU NRS | 2.0 (2.0–2.0) | 2.0 (2.0–2.0) | +0.14 | 0.203 |
| 6-h NRS | 1.0 (1.0–1.0) | 1.0 (1.0–2.0) | +0.16 | 0.395 |
| 24-h NRS | 1.0 (1.0–1.0) | 1.0 (1.0–1.0) | +0.21 | 0.873 |

| | | | | |
|---|---|---|---|---|
| ASA physical status 3 | 1 (3.8) | 4 (8.5) | | 0.649 |
| Hypertension | 4 (15.4) | 7 (14.9) | | 1.000 |
| Diabetes mellitus | 1 (3.8) | 4 (8.5) | | 0.649 |
| PONV | 4 (15.4) | 4 (8.5) | | 0.444 |
| Any rescue analgesic | 9 (34.6) | 4 (8.5) | | 0.009 |
| Early discontinuation | 3 (11.5) | 4 (8.5) | | 0.694 |
| Breast surgery | 3 (11.5) | 11 (23.4) | | 0.352 |
| Pelviscopic surgery | 7 (26.9) | 13 (27.7) | | 1.000 |
| Robot-assisted surgery | 8 (30.8) | 10 (21.3) | | 0.405 |
| Total laparoscopic hysterectomy | 8 (30.8) | 13 (27.7) | | 0.793 |

Values are median (interquartile range) or number (%). Continuous variables were compared with the Mann–Whitney U test and categorical variables with the Fisher exact test. Of 19 characteristics examined, only rescue analgesic use differed significantly, consistent with event logs persisting for patients who used the pump for longer; the cumulative consumption reported in the main text may therefore modestly overestimate the group mean. SMD: standardized mean difference, NRS: numeric rating scale, PACU: post-anesthesia care unit, ASA: American Society of Anesthesiologists, PONV: postoperative nausea and vomiting.

**Figure S1.** Covariate balance before and after inverse-probability-of-treatment weighting.

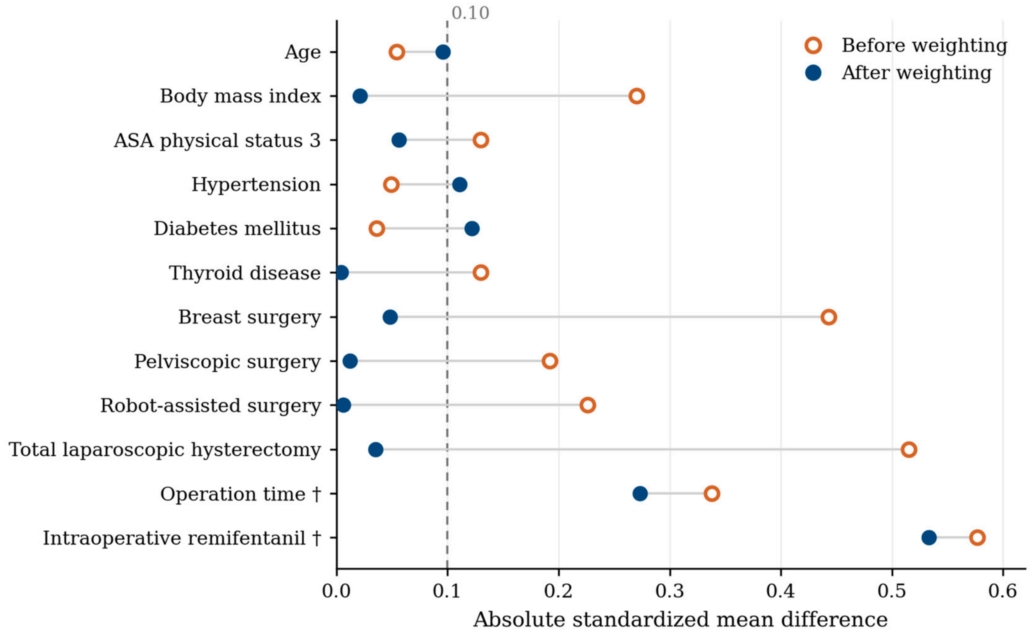


Absolute standardized mean differences for the covariates in the respecified propensity-score model, before (open circles) and after (filled circles) stabilized inverse-probability-of-treatment weighting truncated at the 99th percentile. The dashed line marks the prespecified threshold of 0.10. After weighting the largest absolute standardized mean difference among the model covariates was 0.122 (diabetes mellitus). † Operation time and intraoperative remifentanil dose are shown for reference only; they are determined after treatment allocation and were therefore not included in the propensity-score model.
